# Correction: Factors influencing weight management behavior among college students: An application of the Health Belief Model

**DOI:** 10.1371/journal.pone.0252258

**Published:** 2021-05-20

**Authors:** Maryam Saghafi-Asl, Soghra Aliasgharzadeh, Mohammad Asghari-Jafarabadi

In the Funding section, the grant number from the funder University of South Bohemia Ceske Budejovice is missing. The correct grant number is: 62972.
