# Supplementary material for: Factors influencing weight management behavior among college students: An application of the Health Belief Model
Source: PLoS One. 2020 Feb 7;15(2):e0228058. doi: 10.1371/journal.pone.0228058 (PMC7006943; doi:10.1371/journal.pone.0228058)
Supplement: S2 File — (PDF) [file pone.0228058.s002.pdf]

Questionnaire in Persian

پرسشنامه بررسی عقاید مرتبط با وزن بر اساس مدل باور بهداشتی						
**شدت درک شده**		کاملا مخالفم	مخالفم	نظری ندارم	موافقم	کاملا موافقم
**داشتن اضافه وزن:**	۱- باعث می شود که من احساس نگرانی و استرس کنم.					
	۲- باعث می‌شود که من از نظر دیگران به لحاظ فیزیکی جذاب نباشم.					
	۳- مرا ناراحت و افسرده می‌کند.					
	۴- باعث کاهش عزت نفس من می‌شود.					
	۵- انجام فعالیت بدنی و ورزشی یا تمریناتی که از انها لذت می‌برم را دشوار می‌کند.					
	۶- داشتن خواب کافی را مشکل می‌کند.					
	۷- باعث ایجاد درد در زانوهایم می‌شود.					
	۸- تاثیر نامطلوب بر سلامتی من در سال‌های آتی دارد.					
	۹- خطر ابتلای من به دیابت، فشارخون بالا، سرطان و سایر بیماری‌ها را افزایش می‌دهد.					
	۱۰- پیدا کردن دوست را مشکل می‌کند.					
	۱۱- به‌دست آوردن شغل را به‌دلیل عدم تناسب اندام دچار مشکل می‌کند.					
	۱۲- باعث تمسخر دیگران به‌هنگام برقراری ارتباط اجتماعی با دوستان می‌شود.					
	۱۳- باعث می‌شود نتوانم لباس‌های مورد علاقه ام را بپوشم.					
**حساست درک شده**		کاملا مخالفم	مخالفم	نظری ندارم	موافقم	کاملا موافقم
**من مستعد اضافه وزن داشتن می‌شوم اگر:**	۱- کمتر از ۳۰ دقیقه فعالیت بدنی با شدت متوسط در اکثر روزهای هفته انجام دهم.					
	۲- روزانه یا در اکثر روزهای هفته نوشیدنی‌ها، غذاها و میان‌وعده های حاوی قند و شکر مصرف کنم.					
	۳- روزانه یا در اکثر روزهای هفته غذاها و میان‌وعده‌های سرخ شده مصرف کنم.					
	۴- بیشتر از ۳ بار در هفته در رستوران و بیرون از خانه غذا بخورم.					
	۵- به مقدار غذایی که می‌خورم یا می‌نوشم توجه نکنم.					
	۶- هر دو والدین من یا یکی از آنها بیش وزن یا چاق باشند.					
	۷- سابقه ژنتیکی اضافه وزن یا چاقی داشته باشم.					

**موانع درک شده**	کاملا مخالفم	مخالفم	نظری ندارم	موافقم	کاملا موافقم
۱- نوشیدنی‌ها، غذاها و میان‌وعده‌های کم کالری خیلی گران هستند.					
۲- خرید و تهیه غذاهای سالم وقت زیادی می‌گیرد.					
۳- انجام دادن فعالیت بدنی در اغلب روزها وقت گیر است.					
۴- شغل و تحصیل من مهمتر از داشتن فعالیت بدنی و رژیم غذایی سالم است.					
۵- من انگیزه ای برای داشتن فعالیت بدنی و رژیم غذایی سالم ندارم.					
۶- من از خوردن غذاها و میان‌وعده‌های سرخ شده بیشتر از غذاهای کبابی و بخار پز لذت می‌برم.					
۷- من از خوردن نوشیدنی‌ها، غذاها و میان‌وعده‌های حاوی شکر بیشتر از انواع کم کالری لذت می‌برم.					
۸- من برای داشتن احساس آرامش گرایش به خوردن غذا پیدا می‌کنم.					
۹- من اغلب احساس افسردگی، نگرانی یا استرس می‌کنم و این مساله داشتن فعالیت فیزیکی و رژیم غذایی سالم را دشوار می‌کند.					
۱۰- من نمی‌دانم کجا می‌توانم اطلاعات درستی درباره رسیدن به وزن مناسب و حفظ آن بدست آورم.					
۱۱- من نمی‌دانم چگونه فعالیت فیزیکی را در برنامه روزانه خود قرار دهم.					
۱۲- من نمی‌دانم نوشیدنی‌ها، غذاها و میان‌وعده‌های سالم را از کجا تهیه کنم.					
۱۳- من نمی‌دانم چگونه نوشیدنی‌ها، غذاها و میان‌وعده‌های کم کالری را آماده کنم.					
۱۴- من نمی‌دانم چگونه نوشیدنی‌ها، غذاها و میان‌وعده‌های کم کالری را انتخاب کنم.					

فواید درک شده		کاملا مخالفم	مخالفم	نظری ندارم	موافقم	کاملا موافقم
رعایت رژیم غذایی سالم و فعالیت فیزیکی مناسب می تواند برای من مفید باشد از طریق:	۱- کاهش افسردگی، نگرانی و استرس.					
	۲- کمک به بهتر نمودن تصور بدنی خود.					
	۳- افزایش عزت نفس.					
	۴- بهبود روحیه و خلق.					
	۵- آسان کردن فعالیت های ورزشی که از آن لذت می‌برم.					
	۶- احساس پر انرژی بودن.					
	۷- افزایش شانس من برای داشتن سلامتی فعلی و آتی.					
	۸- بهبود علایم بیماری یا مشکلات سلامتی کنونی.					
	۹- داشتن تناسب فیزیکی برای بهبود عملکرد شغلی و رسیدن به اهداف شغلی و حرفه ای.					
	۱۰- داشتن خواب راحت.					
	۱۱- آسان نمودن انجام فعالیت‌های روزانه.					
	۱۲- فراهم نمودن موقعیت بهتر برای ازدواج.					
	۱۳- داشتن احساس راحتی هنگام حضور در جمع.					

راهنمای عمل		کاملا مخالفم	مخالفم	نظری ندارم	موافقم	کاملا موافقم
من ممکن است تصمیم به داشتن عادات غذایی سالم و فعالیت فیزیکی مناسب داشته باشم اگر:	۱- به آینه نگاه کنم و از بدن خود ناراضی باشم.					
	۲- لباس‌هایم برایم تنگ شود.					
	۳- مشکل سلامتی پیش آید که نیازمند رسیدن به وزن مناسب باشد.					
	۴- بر این باور باشم که دیگران بر اساس وزن من قضاوت نادرستی درمورد من دارند.					
	۵- داشتن وزن مناسب در رسیدن من به اهداف شخصی و حرفه‌ای کمک کند.					
	۶- داشتن وزن مناسب احساس افسردگی، نگرانی یا استرس را در من بهبود بخشد.					
	۷- پزشک، پرستار یا مشاور تغذیه توصیه کند که به وزن مناسب دست یابم.					
	۸- کسی که دوستش دارم به دلیل اضافه وزن یا چاقی دچار مشکل سلامتی شود.					
	۹- یکی از اعضای خانواده و یا دوستان صمیمی ام توصیه کنند که به وزن مناسب دست یابم.					
	۱۰- اطلاعاتی درمورد خطرات سلامتی مرتبط با اضافه وزن یا چاقی در دوره درسی دانشگاهی بدست اورم.					
	۱۱- اطلاعاتی درمورد خطرات سلامتی مرتبط با اضافه وزن یا چاقی از رادیو یا تلویزیون یا شبکه‌های اجتماعی کسب کنم.					
	۱۲- تبلیغات محصول یا خدماتی که برای دستیابی به وزن مناسب کمک کننده باشد را ببینم.					

**خودکارآمدی در رژیم غذایی**	کاملا مخالفم	مخالفم	نظری ندارم	موافقم	کاملا موافقم
۱- من می‌توانم سه وعده غذایی سالم به‌طور منظم مصرف کنم.					
۲- من می‌توانم در وعده های غذایی مقدار متوسطی از غذا مصرف کنم.					
۳- من می‌توانم غذاهای سالم و تازه را بجای غذاهای فراوری شده مصرف کنم.					
۴- من می‌توانم از خوردن شیرینی جات و کیک و شکلات خودداری کنم.					
۵- من می‌توانم از خوردن غذاهای پرچرب مانند غذاهای سرخ شده پرهیز کنم.					
۶- من می‌توانم از مصرف نوشیدنی‌های کربناته مانند کولا خودداری کنم.					
۷- من می‌توانم غذاهای متنوعی مصرف کنم تا از ایجاد عدم تعادل در رژیم غذایی دوری کنم.					
۸- من می‌توانم قبل از خواب از خوردن اجتناب کنم.					
۹- من می‌توانم به آهسته‌گی غذا بخورم حتی زمانی که گرسنه هستم.					
۱۰- من می‌توانم قبل از پرشدن معده ام از خوردن دست بردارم حتی درصورتی که غذا خوشمزه باشد.					
۱۱- من می‌توانم صبح زود بیدار شوم تا صبحانه بخورم.					
۱۲- من می‌توانم زمانی که غذا میخورم از تماشای تلویزیون یا خواندن کتاب خودداری کنم.					
۱۳- من می‌توانم زمانی که اعضای خانواده و یا دوستانم غذا تعارف می‌کنند از خوردن خودداری کنم.					
۱۴- من می‌توانم زمانی که خسته هستم از خوردن خودداری کنم.					
۱۵- من می‌توانم زمانی که گرسنه هستم از خوردن اجتناب کنم.					
۱۶- من می‌توانم زمانی که عصبی هستم از خوردن اجتناب کنم.					
۱۷- من می‌توانم زمانی که افسرده هستم از خوردن اجتناب کنم.					
۱۸- من می‌توانم زمانی که مضطرب یا هیجان زده هستم از خوردن اجتناب نمایم.					
۱۹- من قصد دارم برای کاهش وزن طی شش ماه آینده رژیم غذایی خودرا کنترل کنم.					
۲۰- من قصد دارم برای کاهش وزن طی شش ماه آینده به مشاور تغذیه مراجعه کنم.					
۲۱- من قصد دارم در کلاس‌های کنترل چاقی اگر در دانشگاه برگزار شود شرکت کنم.					

**خودکارآمدی در فعالیت فیزیکی**	کاملا مخالفم	مخالفم	نظری ندارم	موافقم	کاملا موافقم
۱- من می‌توانم روزانه بیشتر از ۱۵ دقیقه پیاده روی کنم.					
۲- من می‌توانم تا زمانی که دچار نفس تنگی نشده‌ام ورزش کنم.					
۳- من می‌توانم در آب و هوای سرد ورزش کنم.					
۴- من می‌توانم در آب و هوای گرم ورزش کنم.					
۵- من می‌توانم همراه با دوستانم بعد از دانشگاه ورزش کنم.					
۶- من می‌توانم بجای تماشای تلویزیون در اوقات فراغت ورزش کنم.					
۷- من می‌توانم بجای آسانسور از پله استفاده کنم.					

**قصد رفتاری مدیریت وزن**	کاملا مخالفم	مخالفم	نظری ندارم	موافقم	کاملا موافقم
۱- من قصد دارم برای کاهش وزن طی شش ماه آینده رژیم غذایی خودرا کنترل کنم.					
۲- من قصد دارم برای کاهش وزن طی شش ماه آینده به مشاور تغذیه مراجعه کنم.					
۳- من قصد دارم در کلاس‌های کنترل چاقی اگر در دانشگاه برگزار شود شرکت کنم.					
۴- من قصد دارم برای کاهش وزن طی شش ماه آینده به‌طور منظم ورزش کنم.					
۵- من قصد دارم برای کاهش وزن طی شش ماه آینده در کلاس یا باشگاه ورزشی شرکت کنم.					
