# Supplementary material for: Factors influencing weight management behavior among college students: An application of the Health Belief Model
Source: PLoS One. 2020 Feb 7;15(2):e0228058. doi: 10.1371/journal.pone.0228058 (PMC7006943; doi:10.1371/journal.pone.0228058)
Supplement: Attachment — Submitted filename: PONE-D-19-22092_reviewer.docx [file pone.0228058.s003.docx]

**PLOS ONE**

### Factors influencing weight management behavior among College Students: An application of the Health Belief Model

--Manuscript Draft--

| **Manuscript Number:** | PONE-D-19-22092 |
| --- | --- |
| **Article Type:** | Research Article |
| **Full Title:** | Factors influencing weight management behavior among College Students: An application of the Health Belief Model |
| **Short Title:** | Factors influencing weight management behavior among College Students |
| **Corresponding Author:** | soghra aliasgharzadeh  Tabriz University of Medical Sciences Tabriz, IRAN, ISLAMIC REPUBLIC OF |
| **Keywords:** | health belief model; weight management; college students; structural equation modeling |
| **Abstract:** | Background  Overweight and obesity have become a significant public health concern in both developing and developed countries. Due to the health implications of weight-reduction behaviors, it is important to explore the factors that predict their occurrence. Therefore, the present study was performed to examine factors affecting the behavioral intention of weight management as well as assess the predictive power of the Health Belief Model (HBM) for body mass index (BMI).  Methods  This cross-sectional study was conducted among 336 female students recruited from dormitories of Tabriz University of Medical Sciences, using quota sampling technique. Data were collected by a structured questionnaire in seven parts (including perceived severity, perceived susceptibility, perceived benefit, perceived barrier, cue to action, self-efficacy in dieting and physical activity, and behavioral intention of weight management), based on the HBM. Structural equation modeling (SEM) was conducted to identify the relationship between HBM constructs and behavioral intention of weight management. Linear regression model were performed to test the ability of the HBM to predict students’ BMIs.  Results  Higher level of perceived threats (sum of perceived susceptibility and severity) (β=0.41, P<0.001), perceived benefits (β=0.19, P=0.009), self-efficacy in exercise (β=0.17, P=0.001), and self-efficacy in dieting (β=0.16, P=0.025) scales was significantly related to greater behavioral intention of weight management. Moreover, perceived threat mediated the relationships between perceived cue to action, perceived benefits, self- efficacy in exercise, and weight management practices. The fit indices of the SEM model seemed acceptable. The final regression model explained approximately 40% of variance in BMI (P<0.001). Additionally, perceived severity, barrier, and self-efficacy in dietary life were the significant variables to predict students’ BMIs.  Conclusions  These findings suggest that health education programs based on the HBM needs to be integrated in preventive health programs and health interventions strategies to ensure adherence and prosperity of the participants. |
| **Order of Authors:** | soghra aliasgharzadeh |
|  | Maryam Saghafi-Asl |
|  | Mohammad Asghari-Jafarabadi |
| **Additional Information:** |  |
| **Question** | **Response** |
| **Financial Disclosure**  Enter a financial disclosure statement that | The author(s) received no specific funding for this work. |

*Powered by Editorial Manager® and ProduXion Manager® from Aries Systems Corporation*

| describes the sources of funding for the work included in this submission. Review the [submission guidelines](http://journals.plos.org/plosone/s/submission-guidelines#loc-financial-disclosure-statement) for detailed requirements. View published research articles from [*PLOS ONE*](http://journals.plos.org/plosone/) for specific examples.  This statement is required for submission and **will appear in the published article** if the submission is accepted. Please make sure it is accurate. |  |
| --- | --- |
| **Unfunded studies**  Enter: *The author(s) received no specific funding for this work.*  **Funded studies**  Enter a statement with the following details:   - Initials of the authors who received each award - Grant numbers awarded to each author - The full name of each funder - URL of each funder website - Did the sponsors or funders play any role in the study design, data collection and analysis, decision to publish, or preparation of the manuscript? - **NO** - Include this sentence at the end of your statement: *The funders had no role in study design, data collection and analysis, decision to publish, or preparation of the manuscript.* - **YES** - Specify the role(s) played. |  |
| * typeset |  |
| **Competing Interests**  Use the instructions below to enter a competing interest statement for this submission. On behalf of all authors, disclose any [competing interests](http://journals.plos.org/plosone/s/competing-interests) that could be perceived to bias this work—acknowledging all financial support and any other relevant financial or non- financial competing interests.  This statement **will appear in the published article** if the submission is accepted. Please make sure it is accurate. View published research articles from [*PLOS ONE*](http://journals.plos.org/plosone/) for specific examples. | The authors have declared that no competing interests exist. |

*Powered by Editorial Manager® and ProduXion Manager® from Aries Systems Corporation*

| **NO authors have competing interests**  Enter: *The authors have declared that no competing interests exist.*  **Authors with competing interests**  Enter competing interest details beginning with this statement:  *I have read the journal's policy and the authors of this manuscript have the following competing interests: [insert competing interests here]* |  |
| --- | --- |
| * typeset |  |
| **Ethics Statement**  Enter an ethics statement for this submission. This statement is required if the study involved:   - Human participants - Human specimens or tissue - Vertebrate animals or cephalopods - Vertebrate embryos or tissues - Field research   Write "N/A" if the submission does not require an ethics statement.  General guidance is provided below. Consult the [submission guidelines](http://journals.plos.org/plosone/s/submission-guidelines#loc-guidelines-for-specific-study-types) for detailed instructions. **Make sure that all information entered here is included in the Methods section of the manuscript.** | This study was approved by the ethics committee of the Tabriz University of Medical Sciences. Informed written consent to participate was obtained from all participants before the start of the study. |

*Powered by Editorial Manager® and ProduXion Manager® from Aries Systems Corporation*

| **Format for specific study types**  **Human Subject Research (involving human participants and/or tissue)**   - Give the name of the institutional review board or ethics committee that approved the study - Include the approval number and/or a statement indicating approval of this research - Indicate the form of consent obtained (written/oral) or the reason that consent was not obtained (e.g. the data were analyzed anonymously)   **Animal Research (involving vertebrate animals, embryos or tissues)**   - Provide the name of the Institutional Animal Care and Use Committee (IACUC) or other relevant ethics board that reviewed the study protocol, and indicate whether they approved this research or granted a formal waiver of ethical approval - Include an approval number if one was obtained - If the study involved *non-human primates*, add *additional details* about animal welfare and steps taken to ameliorate suffering - If anesthesia, euthanasia, or any kind of animal sacrifice is part of the study, include briefly which substances and/or methods were applied   **Field Research**  Include the following details if this study involves the collection of plant, animal, or other materials from a natural setting:   - Field permit number - Name of the institution or relevant body that granted permission |  |
| --- | --- |
|  |  |
| **Data Availability**  Authors are required to make all data underlying the findings described fully available, without restriction, and from the time of publication. PLOS allows rare exceptions to address legal and ethical concerns. See the [PLOS Data Policy](http://journals.plos.org/plosone/s/data-availability) and [FAQ](http://journals.plos.org/plosone/s/data-availability#loc-faqs-for-data-policy) for detailed information. | No - some restrictions will apply |

*Powered by Editorial Manager® and ProduXion Manager® from Aries Systems Corporation*

| A Data Availability Statement describing where the data can be found is required at submission. Your answers to this question constitute the Data Availability Statement and **will be published in the article**, if accepted. |  |
| --- | --- |
| **Important:** Stating ‘data available on request from the author’ is not sufficient. If your data are only available upon request, select ‘No’ for the first question and explain your exceptional situation in the text box. |  |
| Do the authors confirm that all data underlying the findings described in their manuscript are fully available without restriction? |  |
| **Describe where the data may be found in full sentences. If you are copying our sample text, replace any instances of XXX with the appropriate details.**   - If the data are **held or will be held in a public repository**, include URLs, accession numbers or DOIs. If this information will only be available after acceptance, indicate this by ticking the box below. For example: *All XXX files are available from the XXX database (accession number(s) XXX, XXX.*). - If the data are all contained **within the manuscript and/or Supporting Information files**, enter the following: *All relevant data are within the manuscript and its Supporting Information files.* - If neither of these applies but you are able to provide **details of access elsewhere**, with or without limitations, please do so. For example:   *Data cannot be shared publicly because of [XXX]. Data are available from the XXX Institutional Data Access / Ethics Committee (contact via XXX) for researchers who meet the criteria for access to confidential data.*  *The data underlying the results presented in the study are available from (include the name of the third party* | All relevant data are within the manuscript and its Supporting Information files. |

*Powered by Editorial Manager® and ProduXion Manager® from Aries Systems Corporation*

| *and contact information or URL).*   - This text is appropriate if the data are owned by a third party and authors do not have permission to share the data.   * typeset |  |
| --- | --- |
| Additional data availability information: | Tick here if the URLs/accession numbers/DOIs will be available only after acceptance of the manuscript for publication so that we can ensure their inclusion before publication. |

*Powered by Editorial Manager® and ProduXion Manager® from Aries Systems Corporation*

Manuscript	[Click here to download Manuscript manuscript.docx](https://www.editorialmanager.com/pone/download.aspx?id=24878845&amp;guid=aac509c9-dcf5-4457-bea3-1e88438397b8&amp;scheme=1)

***Factors influencing weight management behavior among College Students: An application of the Health Belief Model***

Maryam Saghafi-Asl^1^, Soghra Aliasgharzadeh2* , Mohammad Asghari-Jafarabadi 3

1 Nutrition Research Center, School of Nutrition and Food Sciences, Tabriz University of Medical Sciences, Tabriz, Iran.

2 Student Research Committee, School of Nutrition and Food Sciences, Tabriz University of

Medical Sciences, Tabriz, Iran.

3 Road Traffic Injury Research Center, Tabriz University of Medical Sciences, Tabriz, Iran.

#### * Corresponding author:

E-mail: [Soghra.aliasgharzadeh@gmail.com](mailto:Soghra.aliasgharzadeh@gmail.com)

Phone: 00984133357580

**Running heading:** Factors influencing weight management behavior among College Students

# Abstract

**Background:** Overweight and obesity have become a significant public health concern in both developing and developed countries. Due to the health implications of weight-reduction behaviors, it is important to explore the factors that predict their occurrence. Therefore, the present study was performed to examine factors affecting the behavioral intention of weight management as well as assess the predictive power of the Health Belief Model (HBM) for body mass index (BMI).

**Methods:** This cross-sectional study was conducted among 336 female students recruited from dormitories of Tabriz University of Medical Sciences, using quota sampling technique. Data were collected by a structured questionnaire in seven parts (including perceived severity, perceived susceptibility, perceived benefit, perceived barrier, cue to action, self-efficacy in dieting and physical activity, and behavioral intention of weight management), based on the HBM. Structural equation modeling (SEM) was conducted to identify the relationship between HBM constructs and behavioral intention of weight management. Linear regression model were performed to test the ability of the HBM to predict students’ BMIs.

**Results:** Higher level of perceived threats (sum of perceived susceptibility and sever- ity) (β=0.41, P<0.001), perceived	benefits	(β=0.19, P=0.009), self-efficacy	in exercise (β=0.17, P=0.001), and self-efficacy in dieting (β=0.16, P=0.025) scales was significantly related to greater behavioral intention of weight management. Moreover, perceived threat mediated the relationships between perceived cue to action, perceived benefits, self-efficacy in exercise, and weight management practices. The fit indices of the SEM model seemed acceptable. The final regression model explained approximately 40% of variance in BMI (P<0.001). Additionally, perceived severity, barrier, and self-efficacy in dietary life were the significant variables to predict students’ BMIs.

**Conclusions:** These findings suggest that health education programs based on the HBM needs to be integrated in preventive health programs and health interventions strategies to ensure adherence and prosperity of the participants.

# Introduction

Overweight and obesity have become epidemic rising trends in both developed and developing countries [1-4]. According to estimates by World Health Organization (WHO) in 2016, there were approximately 1.9 billion overweight adults aged 18 years and above from which at least 650 million were obese [5]. The growing trend in the transition from overweight status to obesity often occurs at ages 18–29 years. Obesity is one of the important concerns of health care professionals, as numerous physical and psychological problems including coronary heart disease, diabetes, and several cancers accompany wit it [6-8]. Obesity also imposes enormous financial burdens on both governments and individuals [9]. Several factors contribute to obesity including genetics and behavioral and environmental parameters such as physical activity and dietary behavior [10].

Collegiate period is a critical time for altering physical activity and dietary patterns which lead to gain weight of students during these years [11, 12]. Thus, weight management remains an important health challenge for this population. Several preventive and treatment programs are applied for weight control [13]. However, compliance with weight-loss treatments varies among women for a range of reasons [13, 14]. Previous studies have shown that psychosocial factors such as perceptions about health and obesity and self-efficacy play important roles in the success of weight loss and maintenance programs [15-17].

In order to develop effective weight management interventions for college students, it is important to understand the factors that predict the occurrence of appropriate weight reduction behavior. The Health Belief Model (HBM) is a health-specific social cognitive model that

attempts to predict and explain why individuals change or maintain specific health behaviors [18]. This model assumes that individual involvement in health-related behaviors is determined by understanding six following constructs: *Perceived severity* (an individual's perception of the seriousness and potential consequences of the condition), *Perceived susceptibilit*y (an individual's assessment of their risk of getting a disease or condition), *Perceived benefit* (an individual's beliefs about whether the recommended behavior will reduce the risk or severity of impact), *Perceived barrier* (an individual's assessment of the difficulties and cost of adopting behaviors), *Cue to action* (the internal or external motivations promoting the desired behavior), *Self-efficacy* (an individual's belief about their capabilities to successfully perform a new health behavior). These six constructs provide a conceptual framework for designing both long and short-term health behavior interventions (Fig 1) [18, 19].

#### Fig 1: Theoretical framework of Health Belief Model applied to behavioral intention of weight management.

Several studies have been conducted regarding the effective factors on weight control intention through the HBM [20-23]. Park *et al*. examined factors affecting behavior intention of weight reduction among female middle-school students, using HBM [20]. They found that Perceive threat (a sum of severity and susceptibility), cues to action, and perceived self-efficacy were significantly associated to behavioral intention of weight reduction for all respondents [20]. McArthur et al. tested the predictive power of the HBM (which consisted of perceived susceptibility, perceived severity, perceived benefits, perceived barriers, and cues to action) for body mass index (BMI) among the college student sample [21]. They found significant positive associations between ratings on the perceived susceptibility, perceived barriers, perceived benefits scales and BMI. Findings also revealed significant inverse associations between ratings on the perceived severity, external cues to action scales and BMI [21].

To best of our knowledge, it seems that no research has been conducted on the whole HBM constructs for prediction of weight management of college students. Therefore, the present study was aimed to (1) develop and assess the validity and reliability of the an HBM- based questionnaire for weight management behavior, (2) explore the direct, indirect, and total effects of all of the HBM constructs on weight management behaviors among college students, and (3) determine the predictive power of HBM constructs for BMI of students.

# Methods

## Research design and sampling

This cross-sectional study was conducted in Tabriz, Iran in June, 2018. A total of 336 female students were chosen from dormitories of Tabriz University of Medical Sciences, using quota sampling technique. Data were collected by personal interviews, using a structured questionnaire. Informed consents were obtained from the participants, before the onset of the study.

## Measurement Tool

The first version of the questionnaire used in measuring the HBM variables was derived from Park (2011) and McArthur *et al*. (2017) [20, 21]. Eighty-nine statements were included and represented 8 perceptional and behavioral categories, as follow: 13 questions on perceived severity consisting of 3 subscales (emotional/mental, health, physical health/ fitness, and social professional); 7 questions on perceived susceptibility consisting of 2 subscales (lifestyle and environmental); 14 questions on perceived barriers consisting of 3 subscales (practical concerns, emotional/ mental health, and awareness); 13 questions on perceived benefits including 3 subscales (emotional/ mental health, physical health/ fitness, and social/ professional); 12 questions on cues to action consisting of 2 subscales (internal and external cues to action); 18 questions on self-efficacy in dieting including 2 subscales (Habits and

preferences and Emotional/mental health); 7 questions on self-efficacy in exercise, and 5 questions on behavioral intention of weight management consisting of 2 subscales (dieting and exercising). All statements were rated using a five-point Likert scale ranging from 1 (strongly disagree) to 5 (strongly agree). In order to determine the content validity, ten specialists and professionals (outside the team) in the field of Health and Nutrition were consulted. Then, based on the Lawshe's Table, items with higher values of Content Validity Ratio (CVR) (i.e. higher than 0.62 for 10 people) and Content Validity Index (CVI) (*i.e.* higher than 0.75) were considered acceptable [24]. CVI and CVR showed satisfactory results for each item (CVI range: 0.78–1.00 and CVR range: 0.80- 1.00). Reliability was calculated using internal consistency (Cronbach's Alpha). Alpha coefficients equal to or higher than 0.70 were considered satisfactory [25]. The overall reliability of the instrument based on the Cronbach's alpha, was 0.92. To assess the test-retest reliability of the questionnaire, a subgroup of 30 randomly selected students were asked to repeat the survey after a two-week interval. Intraclass correlation coefficient (ICC) was computed to evaluate the stability over time. ICC indicated excellent agreement (ICC = 0.86).

## Statistical Analysis

Data analyses were conducted using STATA version 12. The characteristics and beliefs of the participants were described, using means (SD) and frequencies (percentages), wherever appropriate. Weight groups were divided into three categories: underweight (BMI<18.5 kg/m^2^), normal weight (18.5≤BMI<25 kg/m^2^), and overweight (BMI≥25 kg/m2). There were few obese students, who were put into the overweight group. Chi-square tests were applied to analyze categorized variables. The mean differences were determined by Kruskal Wallis test. In the case of significant results, Mann-Whitney U test with Bonferroni correction was used to assess the pair-wise comparisons.

Multiple imputation in expectation–maximization (EM) algorithm method was run to manage missing data [26]. Path analysis was used as a tool for structural equation modeling (SEM) to determine the relationship between HBM constructs and behavioral intention of weight management and recognize direct and indirect influence of independent variables toward dependent variables. The magnitude of the relationship was measured by path coefficients and correlations, as standardized estimates. Goodness of fit indices selected for model evaluation were: normed chi-square (χ2/df, values lower than 5 were accepted); comparative fit index (CFI, values greater than 0.90 were accepted); Tacker Lewis index (TLI, values greater than

0.90 were accepted); standardized root mean squared residual (SRMR, values lower than 0.05 were accepted); and root mean square error of approximation (RMSEA, values lower than 0.08 were accepted) [27, 28].

A hierarchical linear regression analysis was performed to estimate the relationships between the HBM scales, demographic characteristic, and BMI. P-Values less than 0.05 were considered as statistically significant.

# Results

## Baseline characteristics

A total of 336 students completed the questionnaires. The mean age of the students was 22.02 (±3.02; range, 18–43) years. Based on self-reported weight and height data, the mean BMI was

22.62 (±3.17; range, 15.63–32.72) kg/m^2^. The baseline characteristics of the participants based

on three weight groups are presented in Table 1. The marital status of the students was significantly different among weight groups (P=0.002). The majority (89.9%) of the students were single.

#### Table 1. Baseline characteristics of the study participants

| Variable | All  (n= 336) | Underweight  (n=28) | Normal weight  (n=236) | Overweight  (n=72) | P-value |
| --- | --- | --- | --- | --- | --- |
| ***Marital status*** |  |  |  |  |  |
| Single | 302(89.9) | 28(9.27)^a^ | 217(71.85)^a^ | 57(18.87)^b^ | 0.002^**^ |
| Married | 34(10.1) | 0(0.00)^a^ | 19(55.88)^a^ | 15(44.12)^b^ |  |
| ***Education level*** |  |  |  |  |  |
| BSc degree | 220(65.48) | 19(8.64)^a^ | 155(70.45)^a^ | 46(20.91)^a^ | 0.670^*^ |
| MSc degree | 36(10.72) | 1(2.78)^a^ | 24(66.67)^a^ | 11(30.55)^a^ |  |
| Ph.D. degree | 80(23.81) | 8(10.00)^a^ | 57(71.25)^a^ | 15(18.75)^a^ |  |
| ***Obese family member*** |  |  |  |  |  |
| Yes | 229(68.15) | 14(6.11)^a^ | 158(69.00)^b^ | 57(24.89)^c^ | 0.004^*^ |
| No | 107(31.85) | 16(14.95)^a^ | 78(72.90)^b^ | 13 (12.15)^c^ |  |
| ***Experience in weight loss behavior*** |  |  |  |  |  |
| Yes | 146(43.45) | 1(0.68)^a^ | 95(60.07)^b^ | 50(34.25)^c^ | <0.001^*^ |
| No | 190(56.55) | 27(14.21)^a^ | 141(74.21)^b^ | 22(11.58)^c^ |  |
| ***Experience of diet therapy*** |  |  |  |  |  |
| Yes | 91(27.08) | 1(1.10)^a^ | 50(54.94)^b^ | 40(43.95)^c^ | <0.001^*^ |
| No | 245(72.92) | 27(11.02)^a^ | 186(75.92)^b^ | 32(13.06)^c^ |  |
| ***Experience of exercise therapy*** |  |  |  |  |  |
| Yes | 156(46.43) | 4(2.56)^a^ | 97(62.18)^b^ | 55(35.26)^c^ | 0.001^*^ |
| No | 180(53.57) | 24(13.33)^a^ | 139(77.22)^b^ | 17(9.44)^c^ |  |
| ***Experience of medical treatment*** |  |  |  |  |  |
| Yes | 20(5.95) | 1(5.00)^ab^ | 8(40.00)^a^ | 11(55.00)^b^ | 0.004^*^ |
| No | 316 (94.05) | 27(8.55)^ab^ | 228(72.15)^a^ | 61(19.30)^b^ |  |
| ***Reason of weight management***  ***behavior*** |  |  |  |  |  |
| Health | 80(29.73) | 3(3.75)^a^ | 51(63.75)^a^ | 26(32.50)^a^ | <0.001^*^ |
| Better appearance | 147(54.65) | 8(5.44)^a^ | 116(78.91)^a^ | 23(15.65)^b^ |  |
| Health and better appearance | 38(14.13) | 0(0.00)^a^ | 16(42.11)^a^ | 22(57.89)^b^ |  |
| Other | 4(1.49) | 0(0.00)^a^ | 4(100.00)^a^ | 0(0.00)^a^ |  |
| ***Socioeconomic status*** |  |  |  |  |  |
| Low | 28(8.33) | 1(3.57)^a^ | 22(78.57)^a^ | 5(17.86)^a^ | 0.064^*^ |
| Middle | 208(61.94) | 19(9.14)^a^ | 146(70.19)^a^ | 43(20.67)^a^ |  |
| High | 100(29.76) | 8(8.00)^a^ | 68(68.00)^a^ | 24(24.00)^a^ |  |

Data are expressed as frequency (percent)

*P value based on Chi-squared test.

**P value base on Fisher’s Exact test.

Values within a row with different superscript letters are significantly different.

There were a significant relationship between family history of obesity and weight status of the student (P=0.004). Approximately, 68 percent of the participants had at least one obese family member. More than half of the students had experience trying to lose weight. This experience differed significantly among weight groups (P<0.001). Most of the students controlled their

diet and exercised to lose their weight. More than half of the students responded that they attempted to manage their weight to improve their appearance, while about one-thirds did so for health. There were significant differences in “the reasons for weight reduction” among under- and normal-weight and overweight groups (P<0.001). The socioeconomic status of the students was not significantly different among weight groups.

## Weight-related beliefs of the participants by weight status

Weight-related beliefs of the students comprising the mean scales and related subscales ratings (SD), and the Cronbach's alpha are presented in Table 2. The mean scores of the 13-item perceived severity of the overweight consequences were 3.26±0.76 for all respondents that showed significant differences among the three groups (P≤0.001). Students in the underweight group showed the highest mean score for perceived severity (3.84±0.57). The beliefs for the physical health/fitness subscale received higher ratings than the other severity subscales (3.44±0.85). Underweight and normal weight students rated the emotional/mental health subscale higher than overweight students (P≤0.001). The mean score of physical health/fitness and social/professional subscales showed significant differences among the three groups (P≤0.001).

#### Table 2. Means and Cronbach α’s for Belief Scales and Subscales

|  | All | Underweight | Normal weight | Over weight | P-value |
| --- | --- | --- | --- | --- | --- |
| **Perceived Severity** |  |  |  |  |  |
| Emotional/mental health subscale (Cronbach α=0.89) | 3.41±0.96 | 3.77±0.85^a^ | 3.50 ±0 .88^a^ | 2.97±1.08^b^ | ≤0.001 |
| Physical health/fitness subscale (Cronbach α=0  .84) | 3.44±0.85 | 4.00±0.55^a^ | 3.52 ± 0.77^b^ | 2.93±1.95^c^ | ≤0.001 |
| Social/professional subscale (Cronbach α  =0.71) | 2.90±0.89 | 3.70±0.62^a^ | 2.93 ± 0.86^b^ | 2.48 ±0.80^c^ | ≤0.001 |
| Total (Cronbach α= 0.90) | 3.26±0.76 | 3.84 ±0.57^a^ | 3.33 ± 0.69^b^ | 2.80±0.81^c^ | ≤0.001 |
| **Perceived Susceptibility** |  |  |  |  |  |
| Lifestyle subscale (Cronbach α = 0.82) | 3.50±0.83 | 3.69 ± 0.68 | 3.53 ±0.80 | 3.27 ± 0.93 | 0.051 |
| Environmental subscale (Cronbach α =  0.72) | 3.37±0.90 | 3.51 ± 0.80 | 3.40 ±0.85 | 3.22±1.03 | 0.286 |
| Total (Cronbach α= 0.84) | 3.46±0.76 | 3.64 ± 0.66 | 3.50 ± 0.71 | 3.26 ±0.88 | 0.075 |
| **Perceived Barriers** |  |  |  |  |  |

| Practical concerns subscale (Cronbach α = 0.78) | 2.91±0.82 | 2.41 ±0.69^a^ | 2.80 ± 0.74^b^ | 3.50 ±0.85^c^ | ≤0.001 |
| --- | --- | --- | --- | --- | --- |
| Emotional/mental health subscale  (Cronbachα=0.71) | 3.10±0.84 | 2.35 ±0.81^a^ | 3.01 ± 0.74^b^ | 3.70±0.79^c^ | ≤0.001 |
| Awareness subscale (Cronbach α =0.90) | 2.84±1.00 | 2.39 ±0.71^a^ | 2.66 ± 0.91^a^ | 3.64±0.99^b^ | ≤0.001 |
| Total (Cronbach α = 0.90) | 2.94±0.75 | 2.39 ±0.59^a^ | 2.81 ± 0.64^b^ | 3.60±0.73^c^ | ≤0.001 |
| **Perceived Benefits** |  |  |  |  |  |
| Emotional/mental health  subscale(Cronbachα=0.85) | 3.89±0.67 | 4.04 ±0.46^a^ | 3.92 ±0.63^a^ | 3.51±0.94^b^ | 0.002 |
| Physical health/fitness subscale (Cronbach α=  0.90) | 3.80±0.65 | 3.87 ± 0.71 | 3.79 ±0.64 | 3.47 ±0.99 | 0.093 |
| Social/professional subscale (Cronbach α=0  .75) | 3.54±0.85 | 3.50 ±0.84 | 3.57 ±0.84 | 3.32 ±1.01 | 0.171 |
| Total (Cronbach α= 0.92) | 3.73±0.67 | 3.87 ± 0.58 | 3.80 ±0.57 | 3.46 ±0.72 | 0.044 |
| **Cue to action** |  |  |  |  |  |
| Internal cues (Cronbach α = 0.85) | 3.57±0.76 | 3.61 ± 0.58 | 3.62 ± 0.70 | 3.40 ±0.96 | 0.512 |
| External cues (Cronbach α =0.86) | 3.41±0.77 | 3.45 ± 0.54 | 3.47 ± 0.72 | 3.21 ±0.97 | 0.121 |
| Total (Cronbach α= 0.90) | 3.49±0.70 | 3.53± 0.49 | 3.54± 0.65 | 3.30± 0.90 | 0.228 |
| **Perceived self-efficacy in dieting** |  |  |  |  |  |
| Habits and preferences subscale  (Cronbachα=0.84) | 3.24±0.66 | 3.72±0.52^a^ | 3.28±0.62^b^ | 2.90±0.69^c^ | ≤0.001 |
| Emotional/mental healt subscale  (Cronbachα=0.84) | 3.20±0.96 | 3.99±0.43^a^ | 3.27±0.96^b^ | 2.66±0.86^c^ | ≤0.001 |
| Total (Cronbach α= 0.88) | 3.22±0.64 | 3.81±0.42^a^ | 3.27±0.58^b^ | 2.82±0.67^c^ | ≤0.001 |
| **Perceived self-efficacy in exercise** |  |  |  |  |  |
| Total (Cronbach α= 0.83) | 3.27±0.79 | 3.23±0.75^ab^ | 3.39 ± 0.71^a^ | 2.90 ± 0.95^b^ | 0.001 |
| **Behavioral intention of weight management** |  |  |  |  |  |
| Diet therapy subscale (Cronbach α= 0.77) | 2.93±0.95 | 3.21±0.66 | 2.86±0.95 | 3.05±1.03 | 0.096 |
| Exercise therapy subscale (Cronbach α= 0.72) | 3.28±0.94 | 3.00±0.95 | 3.31±0.93 | 3.31±0.96 | 0.299 |
| Total (Cronbach α= 0.77) | 3.07±0.78 | 3.13±0.54 | 3.04±0.77 | 3.15±0.90 | 0.544 |

P-values are based on Kruskal-Wallis Test.

Values within a row with different superscript letters are significantly different based on Mann - Whitney U with Bonferroni Correction.

The mean score of the total perceived susceptibility of obesity risk was 3.46±0.76 for all respondents. Students in the underweight group had the highest score (3.64±0.66); however, there were no significant differences among the three groups.

The mean score of the 14-item perceived barriers to adopting healthy eating and physical activity habits were 2.94±0.75 for all respondents that showed significant differences among the three groups (P≤0.001). In addition, students in overweight group showed the strongest perceived barrier (3.60±0.73); followed by students in the normal weight (2.81±0.64), and

underweight (2.39±0.59) group. Beliefs from the emotional/mental health subscale received higher rating than other ones.

The mean score of the 13-item perceived benefits to adopting healthy eating and physical activity habits were 3.73±0.67 for all respondents. There were no significant differences in mean rating on total scale among the three groups. The Emotional/mental health subscale construct received higher rating than other ones.

The mean score of the perceived cues to action for weight management was 3.49±0.70 for all respondents. Normal-weight students had the highest score (3.54±0.65), but there were no significant differences among the three groups. The mean rating of external and internal cues to action were not different among the study groups.

The mean rating on the self-efficacy in dieting was 3.22±0.64 for all respondents that showed significant differences among three groups (P≤0.001). As, students in the underweight group showed the strongest belief about their self-efficacy in dieting (3.81±0.42); followed by students in the normal-weight (3.27±0.58) and overweight group (2.82±0.67).

The mean rating on the self-efficacy in exercise was 3.27±0.79 for all respondents. Students in the normal-weight group had the highest score (3.39±0.71) and indicated significant differences in comparison to those in the overweight group (P≤0.001). But these two groups showed no significant difference, compared to the underweight group.

The mean rating on behavioral intention of weight management was 3.07±0.78 for all respondents. The result showed that students intended to manage their weight by exercising rather than dieting. The mean score of the total behavioral intention of weight management and the two subscales did not demonstrate significant differences among the three groups.

**Path models**

Effects of the final model of HBM constructs on weight management behaviors are displayed in Fig 2. This model was identified given the good fit indices (χ2/df =2.68, CFI = 0.99,

TLI=0.95, RMSEA =0.07, SRMR=0.02) for the all students sample. The model indicated that perceived threats, perceived barriers, perceived benefits, self-efficacy in dieting and self- efficacy in exercise directly affected behavioral intention of weight management. Higher level of aforementioned scales was significantly related to greater behavioral intention of weight management. Moreover, cues to action, perceived benefits and self-efficacy in exercise indirectly affected behavioral intention of weight management through the impact of perceived threats. Tables 3 shows total, direct, and indirect effects of HBM constructs on weight management behavior. Perceived threats and perceived benefits were the greatest predictor of weight loss behaviors with a total correlation coefficient of 0.40 and 0.35, respectively. All of these associations were significant, except for the association of perceived barriers and behavioral intention of weight management.

#### Fig 2. Effects of Health Belief Model constructs on behavioral intention of weight management.

Path coefficients were shown above.

*Significant at 0.05 level.

χ2/df =2.68, CFI = 0.99, TLI=0.95, RMSEA = 0.07, SRMR=0.02.

#### Table 3. The Total, Direct and Indirect Effect of Health Belief Constructs on weight management behavior.

|  | **Direct** | **Indirect** | **Total** |
| --- | --- | --- | --- |
| Perceived threat | .41* | - | 0.41* |
| Perceived benefits | 0.18* | 0.17* | 0.35* |
| Perceived barriers | 0.06 | - | 0.05 |
| Cues to action | - | .10* | 0.10* |
| Self-efficacy in dieting | 0.15* | - | 0.15* |
| Self-efficacy in exercise | 0.17* | 0.04* | 0.21* |
| *Significant at 0.05 level. |  |  |  |

**The HBM as a Predictor for BMI**

Table 4 presents findings from the two-step hierarchical regression analysis constructed to test the ability of the HBM and some of the general characteristics to predict BMIs of the college students. The models were constructed from data provided by all students who responded to the whole HBM scales. When perceived severity, perceived susceptibility, perceived benefits, perceived barriers, cues to action, and self-efficacy in dieting and self-efficacy in exercise were regressed against BMI, the model was highly significant (P<0.001). The first model explained approximately 34% of the variance of the students’ BMIs. Self-efficacy in dieting and perceived severity had an inverse significant association with BMI. Self-efficacy in dieting (β=-1.63, P<0.001), perceived barriers (β=1.18, P<0.001), and perceived severity (β=-1.17, P<0.001) seemed to be the most important among these seven variables. Findings also revealed significant positive associations between ratings on the perceived barriers and BMI. In model two, those demographic variables that had significant correlation with BMI were added to model 1. The inclusion of the age and marital status increased the R^2^, and explained 40% of the variance in the BMI (P<0.001).

#### Table 4. Hierarchical Multiple Regression Analysis for predicting Body Mass Index.

|  | **Model 1** | | | | **Model 2** | | | |
| --- | --- | --- | --- | --- | --- | --- | --- | --- |
| Independent Variable | B | SE | Beta | P- value | B | SE | Beta | P- value |
| Perceived severity | -1.08 | 0.25 | -0.26 | <0.001 | -1.17 | 0.24 | -0.28 | <0.001 |
| Perceived susceptibility | 0.38 | 0.23 | 0.09 | 0.083 | 0.40 | 0.22 | 0.10 | 0.064 |
| Perceived benefits | 0.06 | 0.31 | 0.01 | 0.849 | 0.08 | 0.30 | 0.01 | 0.788 |
| Perceived barriers | 1.30 | 0.23 | 0.31 | <0.001 | 1.18 | 0.22 | 0.28 | <0.001 |
| Cues to action | 0.37 | 0.27 | 0.08 | 0.175 | 0.46 | 0.26 | 0.10 | 0.078 |
| Self-efficacy in dieting | -1.50 | 0.26 | -0.30 | <0.001 | -1.63 | 0.26 | -0.33 | <0.001 |
| Self-efficacy in exercise | 0.21 | 0.21 | 0.05 | 0.336 | 0.31 | 0.21 | 0.08 | 0.129 |
| Age |  |  |  |  | 0.23 | 0.05 | 0.22 | <0.001 |
| Marital status* |  |  |  |  | -0.97 | 0.48 | -0.09 | 0.042 |
| Adjusted R^2^ | 0.34 | | | | 0.40 | | | |

*Reference group was those married. Dependent variable was BMI.

# Discussion

The present study was conducted to investigate the factors influencing behavioral intention by applying the HBM and to estimate the relationships between several belief scales and BMIs of students. Weight loss is usually less successful, despite applying various weight-loss programs, available to the public; once succeeded, the maintenance as well as long-term weight-loss program compliance rates are usually low [29]. Therefore, identification of psychological predictors of weight management could contribute to improve treatment efficacy [15-17].

The present results showed that students with different weight status have different perceptions about obesity and weight reduction behavior. The constructed SEM in this study supported theoretical framework, indicating that health beliefs can directly and indirectly predict student's behavior intention of weight management. In addition, the HBM scales partially predicted the students’ BMIs.

Our ﬁnding showed that the most common weight management methods among students were exercise and dieting. This result is consistent with those of other studies that examined weight- loss practices among university students [30, 31]. Nearly, 55% of the students responded that they attempted to control their weight for better appearance. Our findings are in line with those of other study which indicated that keeping up appearance was the main reason of managing body weight among university students [31]. Socioeconomic conditions of participant were not related to their weight status. Previous studies reported contradictory results considering the association between socioeconomic status and BMI [20, 32-34]. The lack of standard methods of categorizing SES might be the main reason of contradictory [35].

Overweight students in comparison with other groups showed the lower ratings on perceived severity and self-efficacy in dieting and self-efficacy in exercise, but the higher ratings on all subscales of perceived barriers to adopt healthy eating and physical activity habits. The higher ratings on the severity belief scale given by underweight and normal-weight students may have

been motivated them to manage their weight, since individuals make changes if they perceive that their current status could have serious health complications. Some previous studies showed that obese people have less perceived self-efficacy in relation to eating and exercise than non- obese groups [36-38]. Participants’ perceived self-efficacy reflects the confidence in their capacity to perform a new health behavior. A person with a higher level of confidence will be more likely engaged in a specific healthy eating behavior to improve health. In this regard, it was reported that obese Americans are more likely to name several barriers to weight-loss behaviors, compared to non-obese individuals [39]. The results demonstrated that emotional/mental factors, unawareness of healthy food choices, and practical obstacles hamper students to refrain from unhealthy eating behaviors or calorie-dense foods. Moreover, underweight and normal-weight students gave higher, but not significant ratings to perceived susceptibility beliefs than the overweight. Unlike to previous studies, the results suggested that these groups of students may not consider themselves susceptible enough to being overweight to take further action. Moore *et al*. reported that African American normal-weight women reported the same perceived threat of obesity-related diseases as overweight women [40]. In fact, inappropriate perception of own weight and inadequate information about the obesity consequences could lessen the perceived threat of being obese. Students in underweight and normal-weight groups showed the strongest belief about emotional/mental benefits to adopt healthy eating and physical activity habits than others. Differences did not rich the significant level in other subscales of perceived benefit. These results are inconsistent with a prior research [41, 42]. Such ﬁndings suggest that the anticipation of the favorable outcomes of adopting healthy eating habits and engaging in regular physical activity can encourage participants to manage their weight.

In the present study, the constructed SEM helps us better understand the mechanism through which psychosocial factors affect weight management behavior. The results of path analysis

indicated that perceived variables including threat and self-efficacy in dieting have a significant direct effect and perceived benefits and self-efficacy in exercise have a significant direct and indirect effect on predicting weight management behavior. Higher level of the mentioned perceptions further predicted a higher chance of executing behavioral intention of weight management. Perceived threat exerted the greatest influence on behavioral intention of weight management of all respondents, followed by perceived benefit. These results are in agreement with those that suggested perceived benefits, threat, and self-efficacy as the strong predictors of some health behaviors [41-43]. Bishop *et al*. reported that perception of threat and self- efficacy account for considerable amount of the variance in the performance of patient safety practices [43]. Albert Bandura who proposed self-efficacy theory indicated that self-efficacy beliefs contribute to the level and quality of human functioning through cognitive, motivational, affective, and selection processes [44]. When the rate of self-efficacy or person’s confidence of their ability to perform a specific behavior was high, the probability of incorporation in health behavior changes was also increased. O'Connell *et al*. found that dieting benefits were the most powerful predictor of dieting behavior especially for obese adolescent [42]. In the study by Kang *et al.,* perceived benefits were the most important predictor of intention to obesity control among female students [41]. This result suggests that if patients be aware of the benefits of managing weight by dieting and exercising, they might get involved in the programs.

The results showed that perceived barriers to eat healthy foods as well as to undertake regular physical activity could not significantly affect behavior intention of weight management. This result was consistent with the results of some [20, 45], but not other [46, 47] studies which reported that higher perception of the difficulties and cost of performing behaviors are negatively related to a lower likelihood of performing the positive health behaviors. In the present study, the perceived barriers was increased among the students living in dormitories

due to problems such as lack of time, insufficient knowledge, and skills in the preparation of healthy food; that is why, this component failed to justify the behavioral intention of weight management.

In the present research, perceived threat mediates the relationship between cues to action and behavioral intention of weight management. This suggests that external and internal cues would arouse the person’s perceived threat of the risk of obesity by influencing perceived seriousness, susceptibility, or both which led the students to weight management behavior. For instance, the person believes that others judge her unfairly, owing to her weight or an obese family member or a friend, and a serious health problem developed from being obese.

In both regression analysis models, perceived severity, perceived barriers, and self-efficacy in dieting were the significant variables to predict BMI of all respondents. Self-efficacy in dieting seems to be the most significant parameter among the three variables. The final model, in which the demographic variables were added, explained approximately 40% of the variance of students’ BMIs. The results of our study showed that students who assumed to be conﬁdent in their ability to perform the behavior had lower BMIs. This is compatible with previous results showing that obese women scored significantly less than the non-obese on self-efficacy in relation to food [36]. The significant inverse association between perceived severity and students’ BMIs in both of regression models proposed that students who noticed the possible negative physiological, psychological, and social consequences of being obese (*e. g.* chronic disease, mental health problems, difficulties in social relationship) had lower BMI. The significant positive associations between ratings of the perceived barriers scales and students’ BMIs suggested participants who regarded difficulties (*e. g.* lack of time, knowledge, and motivation) and cost of performing behaviors had higher BMI.

There were several worth noting limitations in designing and conducting this study. The main limitation was the cross-sectional, non-experimental design, which provides only a glimpse of

the population at a specific point of time. In addition, only dormitory students of medical sciences were included within this study which confines the generalizability of the findings to all college students. Also, anthropometric data were collected using a self-report nature. Future studies are needed to use HBM to identify the associations between weight-related beliefs of diverse samples and their weight management behaviors. In addition, it would be worthwhile to expand interventional study to investigate the effect of HBM-based educational programs on weigh management in college students or other populations.

# Conclusions

In conclusion, the significant variables to predict behavioral intention of weight management were perceived threat, perceived benefits, self-efficacy in dieting and self-efficacy in exercise, and cues to action. In addition, it was reported that students have different perceptions about obesity and weight reduction behavior by weight status. These results suggested that to ensure adherence and success of the participants in health intervention, it is essential to design and implement health education programs along with dietary approaches. Such programs should emphasize the negative outcomes of obesity, benefit of adopting a healthy lifestyle, increase of self-efficacy in dieting and physical activity and internal and external stimuli’s for college students.

# Acknowledgements

The authors would like to acknowledge Dr. Hossein Karimzadeh, Assistant Professor, Department of Urban Planning of Tabriz University for their assistance with data analysis. Also, the authors thank all participants involved in this study.

# References

1. Bhurosy T, Jeewon R. Overweight and obesity epidemic in developing countries: a problem with diet, physical activity, or socioeconomic status? The Scientific World Journal. 2014;2014.
2. Hales CM, Fryar CD, Carroll MD, Freedman DS, Ogden CL. Trends in obesity and severe obesity prevalence in US youth and adults by sex and age, 2007-2008 to 2015-2016. Jama. 2018;319(16):1723-5.
3. Marques A, Peralta M, Naia A, Loureiro N, de Matos MG. Prevalence of adult overweight and obesity in 20 European countries, 2014. The European Journal of Public Health. 2017;28(2):295-300.
4. Ng M, Fleming T, Robinson M, Thomson B, Graetz N, Margono C, et al. Global, regional, and national prevalence of overweight and obesity in children and adults during 1980–2013: a systematic analysis for the Global Burden of Disease Study 2013. The lancet. 2014;384(9945):766-81.
5. World Health Organization. Obesity and overweight. Available online: [http://www.who.int/news-room/fact-sheets/detail/obesity-and-overweight.](http://www.who.int/news-room/fact-sheets/detail/obesity-and-overweight) (accepted on Nov

2.

6. Frech A. Healthy behavior trajectories between adolescence and young adulthood.

Advances in life course research. 2012;17(2):59-68.

7. Mokdad AH, Serdula MK, Dietz WH, Bowman BA, Marks JS, Koplan JP. The spread of the obesity epidemic in the United States, 1991-1998. Jama. 1999;282(16):1519-22.
8. Vella-Zarb RA, Elgar FJ. The ‘freshman 5’: a meta-analysis of weight gain in the freshman year of college. Journal of American College Health. 2009;58(2):161-6.
9. Tremmel M, Gerdtham U-G, Nilsson PM, Saha S. Economic burden of obesity: a systematic literature review. International journal of environmental research and public health. 2017;14(4):435.
10. Hruby A, Hu FB. The epidemiology of obesity: a big picture. Pharmacoeconomics.

2015;33(7):673-89.

11. Racette SB, Deusinger SS, Strube MJ, Highstein GR, Deusinger RH. Weight changes, exercise, and dietary patterns during freshman and sophomore years of college. Journal of American college health. 2005;53(6):245-51.
12. Karabulut US, Romero Z, Conatser P, Karabulut M. Assessing Overweight/Obesity, Dietary Habits, and Physical Activity in Hispanic College Students. Exercise Medicine. 2018;2.
13. Gill T, King L, Caterson I. Obesity prevention: necessary and possible. A structured approach for effective planning. Proceedings of the Nutrition Society. 2005;64(2):255-61.
14. Kumanyika S. Cultural differences as influences in obesity treatment. Handbook of obesity: Clinical applications. 2008;1:45-68.
15. Lazzeretti L, Rotella F, Pala L, Rotella CM. Assessment of psychological predictors of weight loss: How and what for? World journal of psychiatry. 2015;5(1):56.
16. Faghri P, Simon J, Huedo-Medina T, Gorin A. Effects of self-efficacy on health behavior and body weight. Journal of Obesity & Weight Loss Therapy. 2016;6(6).
17. Hays LM, Finch EA, Saha C, Marrero DG, Ackermann RT. Effect of self-efficacy on weight loss: a psychosocial analysis of a community-based adaptation of the diabetes prevention program lifestyle intervention. Diabetes Spectrum. 2014;27(4):270-5.
18. Rosenstock IM, Strecher VJ, Becker MH. Social learning theory and the health belief model. Health education quarterly. 1988;15(2):175-83.
19. Glanz K, Rimer BK, Viswanath K. Health behavior and health education: theory, research, and practice: John Wiley & Sons; 2008.
20. Park D-Y. Utilizing the Health Belief Model to predicting female middle school students' behavioral intention of weight reduction by weight status. Nutrition research and practice. 2011;5(4):337-48.
21. McArthur LH, Riggs A, Uribe F, Spaulding TJ. Health Belief Model Offers Opportunities for Designing Weight Management Interventions for College Students. Journal of nutrition education and behavior. 2018;50(5):485-93.
22. James DC, Pobee JW, Brown L, Joshi G. Using the health belief model to develop culturally appropriate weight-management materials for African-American women. Journal of the Academy of Nutrition and Dietetics. 2012;112(5):664-70.
23. Zhu DQ, Norman IJ, While AE. Nurses’ self-efficacy and practices relating to weight management of adult patients: a path analysis. International Journal of Behavioral Nutrition and Physical Activity. 2013;10(1):131.
24. Lynn MR. Determination and quantification of content validity. Nursing research. 1986.
25. Cronbach LJ. Coefficient alpha and the internal structure of tests. psychometrika.

1951;16(3):297-334.

26. Allison PD. Missing data techniques for structural equation modeling. Journal of abnormal psychology. 2003;112(4):545.
27. Hu Lt, Bentler PM. Cutoff criteria for fit indexes in covariance structure analysis: Conventional criteria versus new alternatives. Structural equation modeling: a multidisciplinary journal. 1999;6(1):1-55.
28. Hooper D, Coughlan J, Mullen M. Structural equation modelling: Guidelines for determining model fit. Articles. 2008:2.
29. Stubbs J, Whybrow S, Teixeira P, Blundell J, Lawton C, Westenhoefer J, et al. Problems in identifying predictors and correlates of weight loss and maintenance: implications for weight control therapies based on behaviour change. Obesity reviews. 2011;12(9):688-708.
30. Wharton CM, Adams T, Hampl JS. Weight loss practices and body weight perceptions among US college students. Journal of American College Health. 2008;56(5):579-84.
31. Méndez-Hernández P, Dosamantes-Carrasco D, Lamure M, López-Loyo P, Hernández- Palafox C, Pineda-Pérez D, et al. Weight-loss practices among university students in Mexico. International journal of public health. 2010;55(3):221-5.
32. Morgenstern M, Sargent JD, Hanewinkel R. Relation between socioeconomic status and body mass index: evidence of an indirect path via television use. Archives of pediatrics & adolescent medicine. 2009;163(8):731-8.
33. Pawlińska-Chmara R, Wronka I, Suliga E, Broczek K. Socio-economic factors and prevalence of underweight and overweight among female students in Poland. HOMO-Journal of Comparative Human Biology. 2007;58(4):309-18.
34. Wronka I, Suliga E, Pawlińska-Chmara R. Socioeconomic determinants of underweight and overweight in female Polish students in 2009. Anthropologischer Anzeiger. 2012:85-96.
35. Basto‐Abreu A, Barrientos‐Gutiérrez T, Zepeda‐Tello R, Camacho V, Gimeno Ruiz de Porras D, Hernández‐Ávila M. The Relationship of Socioeconomic Status with Body Mass Index Depends on the Socioeconomic Measure Used. Obesity. 2018;26(1):176-84.
36. Richman R, Loughnan G, Droulers A, Steinbeck K, Caterson I. Self-efficacy in relation to eating behaviour among obese and non-obese women. International Journal of Obesity. 2001;25(6):907.
37. Kim NY, Jeong IS, Kim JS. A comparative study on the self-efficacy and health promoting behavior between obese and normal weight middle school students. Journal of Korean Community Nursing. 2001;12(3):828-37.
38. Stutts WC. Physical activity determinants in adults: perceived benefits, barriers, and self efficacy. Aaohn Journal. 2002;50(11):499-507.
39. NEW INSIGHTS INTO AMERICANS’ PERCEPTIONS AND MISPERCEPTIONS OF OBESITY TREATMENTS, AND THE STRUGGLES MANY FACE [internet]. 2016 October [cited	22	Nov	2018].	Available	from: [http://www.norc.org/PDFs/ASMBS%20Obesity/Issue%20Brief%20B_ASMBS%20NORC%](http://www.norc.org/PDFs/ASMBS%20Obesity/Issue%20Brief%20B_ASMBS%20NORC%20Obesity%20Poll.pdf)

[20Obesity%20Poll.pdf.](http://www.norc.org/PDFs/ASMBS%20Obesity/Issue%20Brief%20B_ASMBS%20NORC%20Obesity%20Poll.pdf)

40. Moore SE, Harris C, Wimberly Y. Perception of weight and threat to health. Journal of the national medical association. 2010;102(2):119.
41. Kang Y, Sohn M, Jin K, Kim H, Ohr H, Shin S. Factors influencing weight control behavior and intention of obese children and adolescents. Korean Journal of Preventive Medicine. 1998;31(2):199-214.
42. O'Connell JK, Price JH, Roberts SM, Jurs SG, McKinley R. Utilizing the health belief model to predict dieting and exercising behavior of obese and nonobese adolescents. Health education quarterly. 1985;12(4):343-51.
43. Bishop AC, Baker GR, Boyle TA, MacKinnon NJ. Using the H ealth B elief M odel to explain patient involvement in patient safety. Health Expectations. 2015;18(6):3019-33.
44. Bandura A. Perceived self-efficacy in cognitive development and functioning. Educational psychologist. 1993;28(2):117-48.
45. Garcia K, Mann T. From ‘I wish’to ‘I will’: Social-cognitive predictors of behavioral intentions. Journal of Health Psychology. 2003;8(3):347-60.
46. Huang H-T, Kuo Y-M, Wang S-R, Wang C-F, Tsai C-H. Structural factors affecting health examination behavioral intention. International journal of environmental research and public health. 2016;13(4):395.
47. Deshpande S, Basil MD, Basil DZ. Factors influencing healthy eating habits among college students: An application of the health belief model. Health marketing quarterly. 2009;26(2):145-64.

Figure	[Click here to download Figure fig 1.tif](https://www.editorialmanager.com/pone/download.aspx?id=24878846&amp;guid=5c881a5f-65b3-412e-b634-e520d1860270&amp;scheme=1)

Figure	[Click here to download Figure Fig 2.tif](https://www.editorialmanager.com/pone/download.aspx?id=24878847&amp;guid=cf6b0ec0-913f-45c1-9fb3-43fff752c4f1&amp;scheme=1)
